# Vedolizumab for De Novo Ulcerative Colitis After Kidney Transplantation in a Patient With IgA Nephropathy: A Case Report

**DOI:** 10.1155/crit/3572812

**Published:** 2026-07-14

**Authors:** Monica Cojocaru, Mona Dumbravă, Bogdan Marian Sorohan

**Affiliations:** ^1^ Carol Davila University of Medicine and Pharmacy, Bucharest, Romania, umfcaroldavila.ro; ^2^ Department of Gastroenterology, Fundeni Clinical Institute, Bucharest, Romania, icfundeni.ro; ^3^ Department of Pathology, Fundeni Clinical Institute, Bucharest, Romania, icfundeni.ro; ^4^ Department of Kidney Transplantation, Fundeni Clinical Institute, Bucharest, Romania, icfundeni.ro

**Keywords:** kidney transplantation, treatment, ulcerative colitis, vedolizumab

## Abstract

De novo inflammatory bowel disease (IBD) is a rare but important cause of diarrhea after kidney transplantation (KT). Experience with newer biological agents in the management of IBD among KT recipients remains limited. Vedolizumab is an *α*4*β*7 integrin antagonist that selectively blocks a subset of gastrointestinal‐homing lymphocytes, which limits systemic immunosuppression and, compared to other biologic agents or novel molecules, may reduce the risk of overimmunosuppression in the setting of KT. We report the case of a KT recipient with IgA nephropathy as the underlying cause of chronic kidney disease, who developed de novo ulcerative colitis after KT and was successfully treated with vedolizumab without experiencing adverse reactions during the 29 months of follow‐up.

## 1. Introduction

De novo inflammatory bowel disease (IBD) is a rare but important cause of diarrhea after kidney transplantation (KT) [1, 2]. The incidence of de novo IBD in kidney transplant recipients (KTRs) has been reported to be comparable to that observed after liver transplantation [3]. Transplant‐related immunosuppression, including calcineurin inhibitors, has been suggested to paradoxically contribute to posttransplant IBD [4]. Management of IBD in KTR can be complex and challenging [1]. Experience with newer biological agents in the management of IBD among KTR remains limited [4–6]. Vedolizumab is an *α*4*β*7 integrin antagonist that selectively blocks a subset of gastrointestinal‐homing lymphocytes. Data on the use of vedolizumab in the KTR population are scarce, with only a few available case reports on Crohn′s disease and, to our knowledge, a single case of ulcerative colitis (UC) treated with vedolizumab has been reported to date [7, 8]. Here, we report the case of a KTR with IgA nephropathy (IgAN) as the underlying cause of chronic kidney disease, who developed de novo UC after KT and was successfully treated with vedolizumab without experiencing adverse reactions during the 29 months of follow‐up.

## 2. Case Presentation

A 34‐year‐old male patient with end‐stage renal disease due to IgAN underwent hemodialysis for 9 months before receiving a living‐related KT from his mother, with an HLA compatibility of 8/12. The patient had CMV (cytomegalovirus) IgG‐positive serostatus. Induction immunosuppression was based on antithymocyte globulin, administered at a total dose of 3 mg/kg, in accordance with our protocol for KTR with IgAN. The pretransplant medical history was otherwise unremarkable, except for IgAN, for which the patient had received a short course of prednisone that was discontinued due to a lack of efficacy in the setting of predominantly chronic histopathological lesions on renal biopsy. Pretransplant colonoscopy showed no findings suggestive of IBD or malignancy.

During posttransplant follow‐up prior to the current admission, the patient evolution was favorable without surgical, clinically significant infectious, or rejection episodes. The kidney graft function was stable, with serum creatinine levels stabilizing between 1.15 and 1.39 mg/dL. No abnormalities were detected on urinalysis, tacrolimus trough levels were within the target therapeutic range for the posttransplant period, and anti‐HLA antibodies were absent. In addition, periodic virological monitoring showed undetectable CMV and BK virus (BKV) viremia.

**Figure 1:**
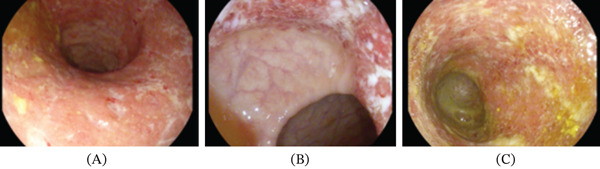
(A) Colonoscopy revealing granular, edematous mucosa with erosions and touch friability, extending up to 40 cm from the anal verge; (B) sharp demarcation observed between the affected and normal mucosa; and (C) follow‐up colonoscopy at 29 months showing endoscopic remission.

**Figure 2:**
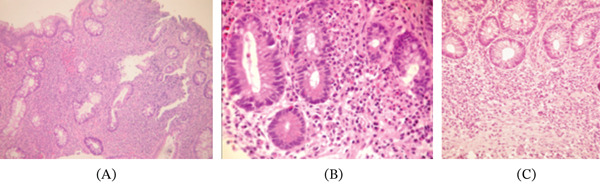
(A–C) Histopathological examination showed moderately distorted glandular architecture with irregular, focally branched crypts, diffuse active chronic inflammation, frequent cryptitis, and crypt microabscesses, basal plasmacytosis, and areas of erosion with superficial fibrinous exudate. Paneth cell metaplasia was present, with no granulomas or evidence of cytomegalovirus infection. Overall, the findings were consistent with chronic inflammatory bowel disease, compatible with UC (Nancy histological index 4).

Eleven months after KT, he was admitted with progressively worsening diarrhea over the preceding 2 weeks, initially mucus‐containing and subsequently bloody, reaching up to 10 stools per day at presentation. The episode was associated with severe abdominal cramps, fatigue, and a weight loss of 5 kg. There was no recent history of gastrointestinal disease or travel. At admission, the patient was on immediate‐release tacrolimus 2 mg twice daily, mycophenolate sodium 360 mg twice daily, prednisone 5 mg/day, oral pH‐dependent release budesonide 3 mg/day administered according to our center′s protocol for the prevention of recurrent IgAN after KT, and candesartan 8 mg/day. Laboratory tests at admission showed mild anemia (hemoglobin 9.9 g/dL), normal leukocytes (7480 cells/*μ*L) and platelets (332,000 cells/*μ*L), increased CRP (9 mg/L), normal serum albumin (4.7 g/dL), and stable kidney function (serum creatinine 1.23 mg/dL, urea 38 mg/dL) without urinalysis abnormalities. Tacrolimus level was within the target therapeutic range (6.3 ng/mL) (Table 1). A comprehensive stool evaluation was performed to exclude infectious causes of colitis in a posttransplant setting. Stool cultures for bacterial pathogens including *Salmonella*, *Shigella*, *Campylobacter*, *Escherichia coli*, and *Clostridium difficile* were negative. Stool viral PCR testing for adenovirus, norovirus, and rotavirus was negative, as was stool examination for ova and parasites. Fecal calprotectin levels were elevated (195 *μ*g/g), which was consistent with active intestinal inflammation. Quantitative blood PCR did not reveal any evidence of CMV DNAemia. Moreover, mycophenolate‐induced colitis was considered in the differential diagnosis. Colonoscopy revealed lesions consistent with left‐sided UC, classified as an endoscopic Mayo score of 2. (Figures 1A–B and 2A–C).

**Table 1:** Patient evolution.

	UC Dx	1 mo	2 mo (vedolizumab start)	3 mo	7 mo∗	11 mo∗∗	12 mo∗∗∗	15 mo	18 mo	24 mo	29 mo^a^
sCr (mg/dL)	1.23	1.28	1.12	1.11	1.2	1.35	1.16	1.27	1.37	1.24	1.3
WBC (cells/*μ*L)	7480	—	—	—	7800	14,350	8000	—	—	—	9000
Hb (g/dL)	9.9	—	—	—	11.9	9.5	11	—	—	—	13.6
PLT (cells/*μ*L)	332,000	—	—	—	360,000	435,000	300,000	—	—	—	300,000
CRP (mg/L)	9	—	—	—	3	33.9	2	—	—	—	0.82
sAlbumin (g/dL)	4.7	—	—	—	4.3	3.9	4.3	—	—	—	5
Fecal calprotectin(*μ*g/g)	195	—	—	—	40	300	78	—	—	—	38
TAC level (ng/mL)	6.3	5	5	5.4	6.5	5.87	6.35	4.5	6.18	7	5.8
Mycophenolate sodium dose	360 mg bid					180 mg bid	360 mg bid				

*Note:* Single asterisk “∗” denotes clinical, biological, and endoscopic remission of UC. Double asterisks “∗∗” mean *Clostridium difficile* infection and UC relapse. Triple asterisks “∗∗∗” indicate UC remission.

Abbreviations: bid, twice daily; CRP, C reactive protein; Hb, hemoglobin; mo, months; PLT, platelets; sAlbumin, serum albumin; sCr, serum creatinine; TAC, tacrolimus; UC, ulcerative colitis; WBC, white blood cells.

^a^ Last follow‐up: clinical, biological, and endoscopic remission of UC.

The initial therapy consisted of oral and rectal 5‐aminosalicylic acid (5‐ASA) associated with 30 mg of oral prednisone. Owing to a lack of response, intravenous vedolizumab was initiated at a dose of 300 mg at Weeks 0, 2, and 6, followed by maintenance dosing every 2 months. Concomitantly, the patient received 5‐ASA foam therapy and continued budesonide 3 mg daily for IgAN recurrence prophylaxis. After 5 months of treatment, clinical, biological, and endoscopic remission were obtained (Table 1). Four months later, the patient developed severe diarrhea, with up to 10 stools per day, some with a bloody appearance, and abdominal pain. *C. difficile* infection was confirmed, prompting mycophenolate dose reduction and the initiation of oral vancomycin. Colonoscopy showed lesions compatible with extensive UC relapse up to the transverse colon (Mayo 3), leading to vedolizumab dose escalation to 300 mg every month. Thereafter, the patient′s clinical course was favorable, achieving sustained clinical, biological, and endoscopic remission throughout the subsequent 20‐month follow‐up period (Table 1 and Figure 1C). The kidney graft function remained stable during the entire follow‐up period, with no abnormalities detected on urinalysis (Table 1). In addition, no CMV or BKV reactivation was observed during this period.

## 3. Discussions

IBD should be included in the differential diagnosis of persistent or unexplained diarrhea after KT. Although de novo IBD following solid organ transplantation is considered uncommon, its true prevalence may be underestimated. Ogata et al. [3] reported that the incidence of de novo IBD after KT may be comparable with that observed after liver transplantation. Data on the use of biologic therapies in this setting remain scarce, especially among KTR [5]. We bring into discussion the case of a KTR who developed de novo UC following KT, and in whom remission was successfully achieved with vedolizumab. Evidence regarding the use of vedolizumab for the treatment of IBD, especially UC, in KTR is limited. In a case series study including three KTRs with Crohn′s disease, vedolizumab was associated with improvement in gastrointestinal symptoms, stable graft function, stable tacrolimus trough levels, and fewer acute kidney injury episodes [7]. Conversely, a case report described vedolizumab‐associated acute interstitial nephritis leading to acute kidney injury in a KTR with UC [8].

In our case, the choice of vedolizumab was guided by moderate disease activity, lack of response to standard treatment, and its gut‐selective mechanism of action, which limits systemic immunosuppression and may reduce the risk of overimmunosuppression in the setting of KT, compared to other biologic agents, such as anti–TNF‐alfa or novel molecules [5, 9]. Vedolizumab successfully induced the clinical, biological, and endoscopic remission of UC. The drug was well tolerated, with no attributed adverse events or significant drug interactions even after dose escalation. The patient experienced a UC flare associated with a *C. difficile* infection. *C. difficile* infection is more frequently observed in patients with UC and is recognized as a potential trigger for disease relapse. After appropriate antimicrobial therapy and escalation of vedolizumab dosing, clinical and endoscopic remission were regained and sustained. Although cases of vedolizumab‐associated acute interstitial nephritis have been reported, no such complications were observed in our patient [10]. Kidney graft function remained stable throughout the follow‐up period, with no episodes of acute kidney injury.

In our patient, UC developed de novo after KT in the context of pre‐existing IgAN despite prophylactic low‐dose oral budesonide for IgAN recurrence. The association between IgAN and UC has been described previously. Notably, nontransplant IgAN patients have a 2.6‐fold increased risk of developing UC. Conversely, chronic intestinal inflammation in UC may trigger IgAN onset or relapse of IgAN [11]. UC and IgAN share common pathophysiological mechanisms characterized by dysregulated mucosal immunity involving aberrant T‐ and B‐lymphocyte activation within the gut‐associated lymphoid tissue. Intestinal inflammation promotes excessive and aberrantly glycosylated IgA production through altered T cell‐dependent B cell responses, thereby linking colonic immune activation to renal immune complex deposition [12]. In this context, gut‐selective immunomodulation may be particularly relevant in IgAN. By blocking *α*4*β*7 integrin–mediated lymphocyte trafficking to the intestinal mucosa, vedolizumab limits pathogenic T and B cell recruitment, potentially reducing the intestinal immune stimulus responsible for the overproduction of galactose‐deficient IgA. This mechanism may offer theoretical benefits in modulating the mucosal component of IgAN, while preserving systemic immune competence and reducing the risk of disease recurrence after KT.

## 4. Conclusion

In conclusion, we report a case of de novo UC after KT in a recipient with underlying IgAN that was successfully treated with vedolizumab, with a good safety profile, stable graft function, and no clinical evidence of recurrent IgAN. IBD should be considered in the differential diagnosis of diarrhea in KTR, even though it represents a relatively uncommon cause. The management of UC with gut‐selective biologic therapy could constitute an effective strategy that minimizes the risk of overimmunosuppression in KTR. In KTR with UC and IgAN, the potential impact of gut‐selective modulation of intestinal inflammation with vedolizumab on the risk of IgAN recurrence after KT remains a hypothesis that warrants further evaluation.

**Nomenclature**
5‐ASA: 5‐aminosalicylic acid
BKV: BK virus
CKD: chronic kidney disease
IBD: inflammatory bowel disease
IgAN: IgA nephropathy
KT: kidney transplantation
KTR: kidney transplant recipients
UC: ulcerative colitis

## Author Contributions

Monica Cojocaru contributed to formal analysis, investigation, writing original draft, and writing review and editing. Mona Dumbravă contributed to data curation, software, writing original draft, and writing review and editing. Bogdan Marian Sorohan contributed to conceptualization, supervision, writing original draft, and writing review and editing.

## Funding

No funding was received for this manuscript.

## Ethics Statement

Written informed consent was obtained from the patient for publication of this case report and any accompanying images. All procedures performed were in accordance with the ethical standards of the institutional and national research committee and with the 1964 Helsinki Declaration and its later amendments.

## Conflicts of Interest

The authors declare no conflicts of interest.

## Data Availability

The data that support the findings of this study are available from the corresponding author upon reasonable request.
